# Supplementary material for: Dexamethasone-Induced Insulin Resistance Attenuation by Oral Sulfur–Oxidovanadium(IV) Complex Treatment in Mice
Source: Pharmaceuticals (Basel). 2024 Jun 10;17(6):760. doi: 10.3390/ph17060760 (PMC11206843; doi:10.3390/ph17060760)
Supplement: Supplementary file 1 [file pharmaceuticals-17-00760-s001.zip › pharmaceuticals-3027971-supplementary.pdf]

Dexamethasone-induced insulin resistance attenuation by oral sulfur-oxidovanadium(IV) complex treatment in mice

Eucilene K. Batista, Lidiane M.A. de Lima, Dayane A. Gomes, Debbie C. Crans*, Wagner E. Silva, Mônica F. Belian* and Eduardo C. Lira

SUPPLEMENTARY MATERIAL

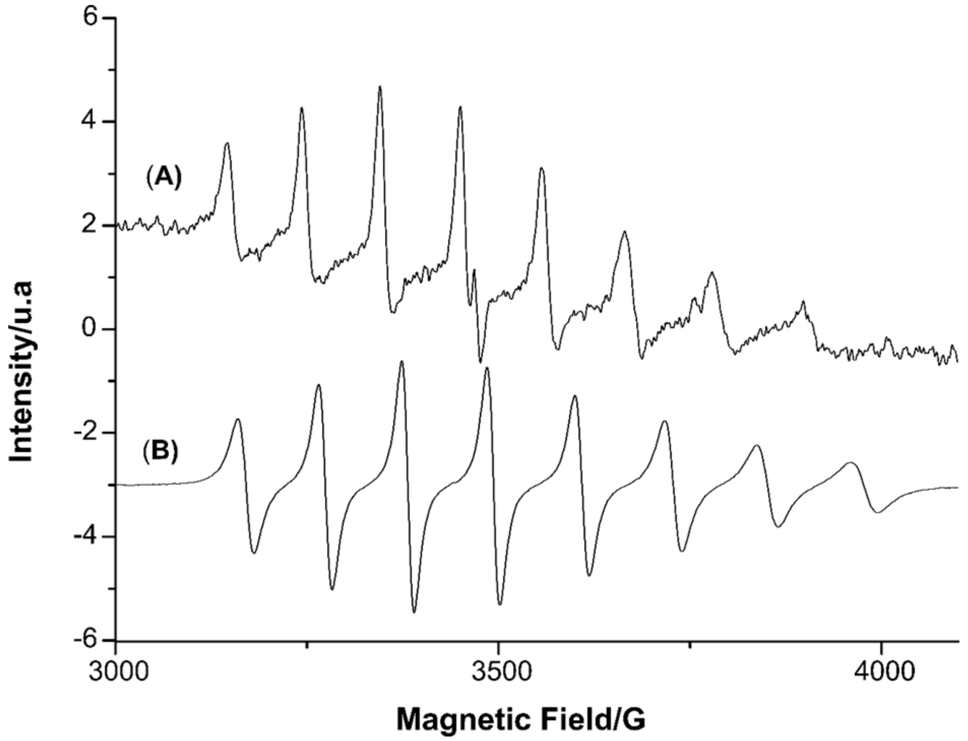

Figure S1. EPR spectrum of the (A) [$V^{IV}O$(octd)] complex (2 mmol L^{-1}; g_{iso} = 1.954) and (B) $VOSO_4$. (50 mmol L^{-1}; g_{iso} = 1.960), in DMSO. The eight-line patterns refer to vanadium nuclear hyperfine couplings of oxidovanadium(IV) compounds (^{51}V, I = 7/2).

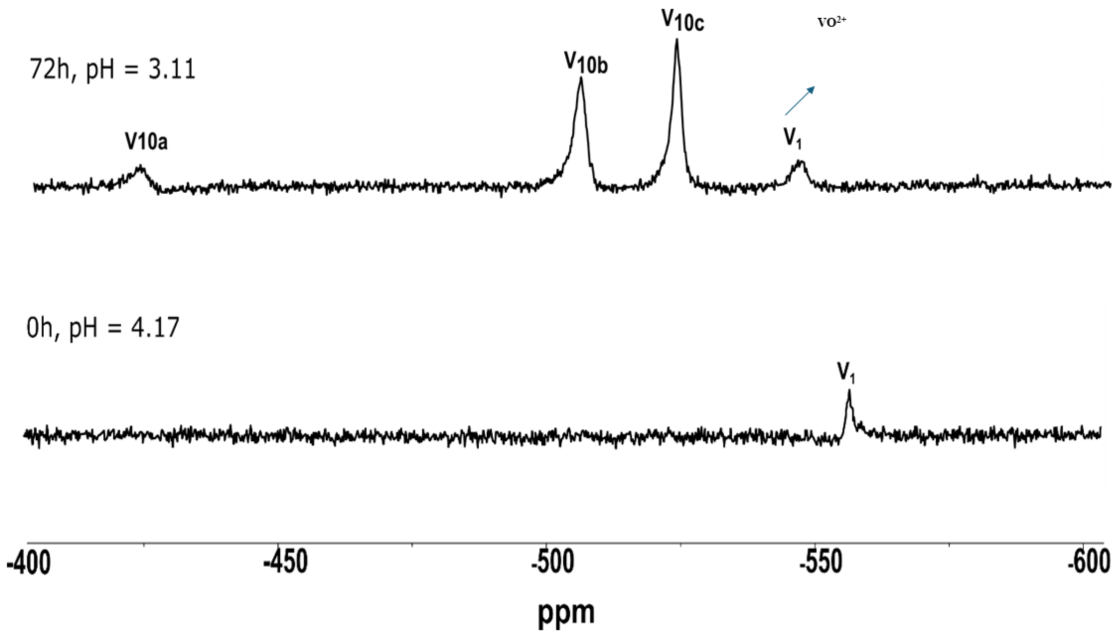

Figure S2. ^{51}V NMR of the [$V^{IV}O$(octd)] complex (2 mmol L^{-1}) at 0 h and 72 h.
